# Computer-Assisted Annotation of Digital H&E/SOX10 Dual Stains Generates High-Performing Convolutional Neural Network for Calculating Tumor Burden in H&E-Stained Cutaneous Melanoma

**DOI:** 10.3390/ijerph192114327

**Published:** 2022-11-02

**Authors:** Patricia Switten Nielsen, Jeanette Baehr Georgsen, Mads Sloth Vinding, Lasse Riis Østergaard, Torben Steiniche

**Affiliations:** 1 Department of Pathology, Aarhus University Hospital, Palle Juul-Jensens Boulevard 35, DK-8200 Aarhus, Denmark; 2 Department of Clinical Medicine, Aarhus University, Palle Juul-Jensens Boulevard 82, DK-8200 Aarhus, Denmark; 3 Center of Functionally Integrative Neuroscience, Aarhus University Hospital, Palle Juul-Jensens Boulevard 99, DK-8200 Aarhus, Denmark; 4 Department of Health Science and Technology, Aalborg University, Fredrik Bajers Vej 7E, DK-9220 Aalborg, Denmark

**Keywords:** deep learning, artificial intelligence, digital pathology, melanoma, immunohistochemistry, H&E, SOX10, IHC-supervised annotation, digital multiple stains, tumor burden

## Abstract

Deep learning for the analysis of H&E stains requires a large annotated training set. This may form a labor-intensive task involving highly skilled pathologists. We aimed to optimize and evaluate computer-assisted annotation based on digital dual stains of the same tissue section. H&E stains of primary and metastatic melanoma (*N* = 77) were digitized, re-stained with SOX10, and re-scanned. Because images were aligned, annotations of SOX10 image analysis were directly transferred to H&E stains of the training set. Based on 1,221,367 annotated nuclei, a convolutional neural network for calculating tumor burden (CNN_TB_) was developed. For primary melanomas, precision of annotation was 100% (95%CI, 99% to 100%) for tumor cells and 99% (95%CI, 98% to 100%) for normal cells. Due to low or missing tumor-cell SOX10 positivity, precision for normal cells was markedly reduced in lymph-node and organ metastases compared with primary melanomas (*p* < 0.001). Compared with stereological counts within skin lesions, mean difference in tumor burden was 6% (95%CI, −1% to 13%, *p* = 0.10) for CNN_TB_ and 16% (95%CI, 4% to 28%, *p* = 0.02) for pathologists. Conclusively, the technique produced a large annotated H&E training set with high quality within a reasonable timeframe for primary melanomas and subcutaneous metastases. For these lesion types, the training set generated a high-performing CNN_TB_, which was superior to the routine assessments of pathologists.

## 1. Introduction

The recent introduction of deep learning for the image analysis of histopathological stains has revolutionized the field of digital pathology. This machine learning technique enables the extraction of high-level information from whole slide images (WSI) through artificial neural networks, which are multi-layered mathematical models inspired by the human brain [1]. A shift towards computer-assisted diagnosis is thus starting to emerge, which has been considered of key importance to facilitate accurate, objective, and time-efficient diagnostic procedures in pathology [2].

Within recent decades, numerous automated procedures with handcrafted algorithms have been proposed. They have mostly been aimed at immunohistochemistry (IHC) that highlights specific biomarkers of interest—for instance, Ki67 or hormone receptors in breast cancer [3,4]. In contrast to IHC, the traditional H&E stain only depicts a general overview of the tissue sample with very similar colorations of most cell types, which complicates image analysis substantially. Accordingly, only handcrafted procedures based on IHC have been approved for diagnosis by the Food and Drug Administration (FDA) or accredited with the European Conformité Européenne (CE) mark so far [5]. 

Yet, H&E is the most widely used stain in medical diagnosis and highly preferred by most pathologists as the initial routine stain because of its unique ability to recapitulate tissue morphology. Furthermore, the stain is easy, fast, and cheap to perform, and thus available at all pathology departments; opposite, for instance, IHC. Consequently, implementation of automated procedures for H&E stains holds great promise for clinical pathology in order to improve efficiency of routine diagnosis, while maintaining the same or even a better diagnostic quality. 

To date, many areas of pathology have been associated with high intra and interobserver variability [6,7,8,9], which reduces the pathologists’ diagnostic performance. One example is the calculation of tumor burden, commonly a prerequisite for many molecular tests [10,11], e.g., BRAF (v-raf murine sarcoma viral oncogene homolog B1) mutational assays in cancer patients. 

In patients with advanced melanoma, approximately one-half harbor a mutation in the BRAF gene, and they experience significant long-term treatment benefit from targeted therapy with BRAF or MEK (mitogen-activated protein kinase) inhibitors. Molecular testing for BRAF mutations are thus of high priority to determine the course of therapy in these patients [12]. Yet, to ensure sensitivity, molecular testing, e.g., with PCR or next-generation sequencing (NGS), requires a predefined tumor-cell content of the included formalin-fixed, paraffin-embedded (FFPE) tissue [10]. Pathologists thus make a semi-quantitative measure (termed eyeballing) of the percentage level of tumor-cell nuclei relative to all nuclei on H&E stains prior to analysis. If the pathologist’s percentage level is low, it is advisable to macrodissect the FFPE section to increase tumor-cell content. Samples with a low tumor-cell content may still be analyzed, but the result is associated with a known reduced sensitivity. Accordingly, an accurate tumor burden is important for an accurate interpretation of the molecular result. 

**Figure 1:**
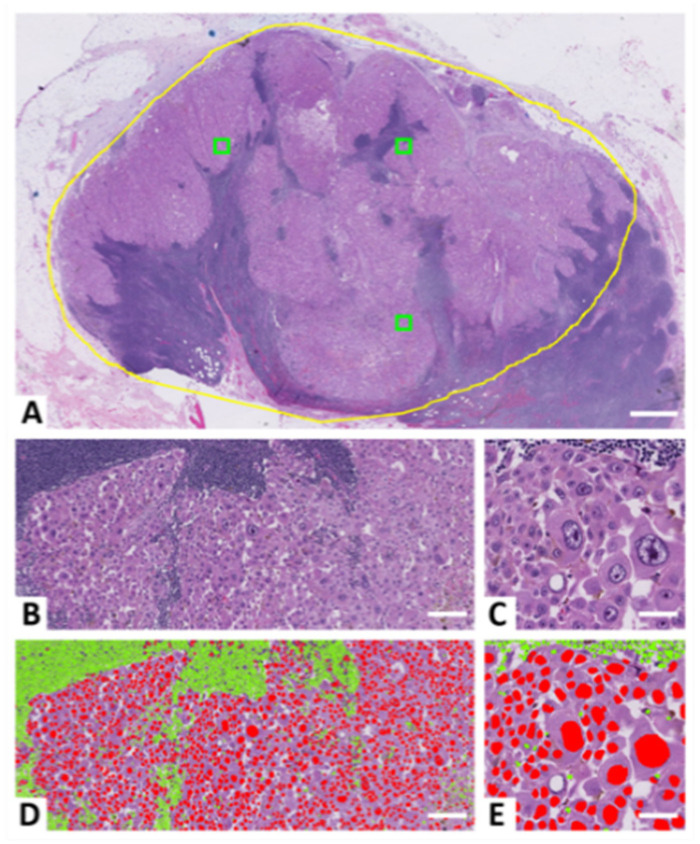
H&E-stained lymph-node melanoma metastasis. The pathologist’s eyeballed tumor burden was 70%; yet, the mutant-allele frequency of next-generation sequencing was 10%. The large difference in nuclei size between normal and tumor cells may have caused the difference, in addition to inaccuracies related to the technician’s manual macrodissection of the tumor. (**A**) Tumor outline (yellow) used for tumor burden calculation and macrodissection, including 0.05 mm^2^ squares (green) of systematic-random sampling, which were used to compare and evaluate study procedures; scalebar 1000 µm. (**B**,**C**) Large melanoma cells and small normal cells; mostly lymphocytes. (**D**,**E**) Automated nuclei detection and calculation of tumor burden by convolutional neural network; scalebars 125 µm and 50 µm, respectively.

Most importantly, if the pathologist overestimates the tumor burden (Figure 1A–C) and macrodissection is consequently left undone, the risk of false-negative results increases, which may leave a melanoma patient without the potential advantages of BRAF or MEK inhibitors. Alternatively, a diagnostic adjunct based on a neural network may guide the pathologist to a more accurate measure of the tumor burden (Figure 1D,E) [13].

In addition to melanoma, tumor burden is also eyeballed by pathologists in other cancer types, e.g., colon cancer [14] and lung cancer prior to treatment decisions [10]. Furthermore, the possibilities of targeted therapy and personalized medicine are steadily increasing, and molecular pathology is thus becoming more and more important. In many cases, the growing number of tests that detect specific gene mutations or genetic abnormalities will possibly need to be accompanied by a tumor burden calculation [10]. 

To date, countless neural networks have been developed or are under development for diagnostic or prognostic purposes in pathology. Often, the performance of the neural network is equal or superior to the evaluation of pathologists [15,16]. Accordingly, applications aimed at H&E stains have already been FDA- or CE-approved for cancer detection in prostate cancer and metastasis detection in lymph nodes of colorectal and breast adenocarcinoma [5]. Sufficient performance of the artificial neural network, however, often depends on a large training set annotated at either image or pixel level. Especially for semantic segmentation of H&E stains because differences between objects of interest may be subtle. In a setup using fully supervised learning, this annotation process forms a very labor-intensive and cumbersome task that frequently involves highly skilled pathologists.

In recent times, the concept of weakly supervised learning has, however, been introduced. However, even though this technique holds great promise, a very large number of slides often needs to be included, and especially many negative or clean slides (without the object of interest) are essential [17]. For the semantic image segmentation of nuclei on H&E stains, weakly supervised learning with initial annotation with partial-points has very recently showed encouraging results, but many manual annotations were still performed [18]. In cases of advanced semantic segmentation, e.g., differentiation of normal-cell and tumor-cell nuclei for the calculation of tumor burden in melanoma, the need for a vast amount of pixel-level annotations still seems evident because their difference in appearance may be subtle [19].

**Figure 2:**
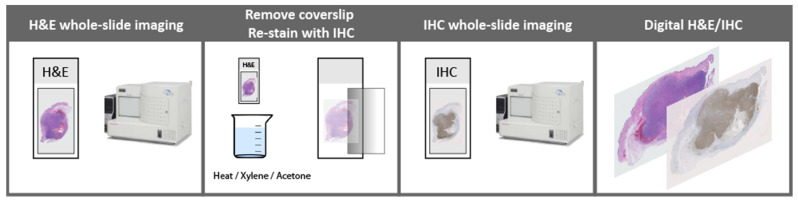
Digital H&E/IHC dual staining of the same tissue section. Initially, the H&E stain is digitized. Then, the glass coverslip is removed. This may be conducted chemically or by heat. Afterwards, chromogenic immunohistochemistry is performed directly on the H&E glass slide and re-scanned. Finally, the two digital images of H&E and immunohistochemistry are superimposed to form a digital dual stain.

Another approach to reduce or eliminate manual annotations is IHC-supervised learning, which a few studies have utilized [20,21,22,23], after the recent introduction of digital dual stains (superimposed WSI) of the same tissue section (Figure 2) [24]. In this technique, pixel-level annotations of IHC image analysis are readily assigned to the corresponding H&E stain. 

Using the melanocytic marker SOX10, Jackson et al. automatically annotated H&E stains of various lesion types (*n* = 12), though mostly primary and metastatic cutaneous melanomas, in order to discriminate nuclei of SOX10-positive and SOX10-negative cells. They state that their lesion and tissue diversity may have improved generalizability of their final CNN, but perhaps at the cost of accuracy. Additionally, a poor nuclear overlap was reported in 34% of their superimposed subimages, which they attribute to warping caused by the washout of H&E. Furthermore, the brown chromogen 3,3′-Diaminobenzidine (DAB) was utilized, which is less useful for melanomas because they often include various amounts of pigmentation and many melanophages; hence, many false-positive annotations will occur in an automatically annotated training set with DAB, unless slides are manually reviewed and edited; a time-consuming and cumbersome task. In an independent test set (*n* = 7), the CNN was evaluated manually, in which most melanocytic cells were correctly classified but false-positive results occurred in lymphocytes and keratinocytes. Rarely, melanoma cells were classified as normal cells [21]. Their study of this method was, however, unaimed at the specific calculation of tumor burden in cutaneous melanoma, and a neural network based solely on such lesion types remains to be developed and explored in a clinical setting.

This study aimed to optimize and evaluate automated, IHC-supervised annotation of tumor-cell and normal-cell nuclei in primary and metastatic cutaneous melanoma based on digital H&E/SOX10 dual stains of the same tissue section, but without H&E washout prior to IHC. Specifically, the performance of IHC-supervised annotation procedures was compared for (1) the use of a brown and a red chromogen, (2) procedures based on either conventional handcrafted algorithms or CNNs, and (3) primary versus metastatic lesions. Finally, in a clinical setting, the CNN for calculating tumor burden (CNN_TB_) based on the best-performing annotation technique was compared with manual annotations, stereological counts, and the performance of routine pathologists. 

## 2. Materials and Methods

### 2.1. Specimens

FFEP blocks were included from patients diagnosed with cutaneous melanoma or cutaneous metastatic melanoma, that is, 30 excised primary melanomas and 47 melanoma metastases, including 32 surgical resections and 15 core needle biopsies.

Lesions were randomly collected from two previous research studies (*n* = 51) that included patients diagnosed between 2001 and 2014 at various pathology departments in Denmark [25,26]. Furthermore, all melanoma patients with a BRAF mutation detected by NGS between 2018 and 2021 at the Department of Pathology, Aarhus University Hospital, (*n* = 26) were included [13]. Accordingly, their tumor burden of routine diagnosis estimated by a pathologist and the mutant-allele frequency of NGS were available from their pathology reports.

### 2.2. Digital H&E/IHC Dual Staining of the Same Tissue Section

The patients’ routine H&E stain used for the tumor burden evaluation was digitized (Figure 2). Then, the glass coverslip was removed by heat (heat plate, 180 °C, seconds to minutes) and slides were placed in xylene (5 min). After re-hydration, H&E glass slides were re-stained with chromogenic IHC, and slides were digitized. Whole slide images of H&E and IHC were subsequently superimposed to form digital dual stains in Visiopharm Integrator System 2020.08 (VIS; Visiopharm A/S, Hørsholm, Denmark). 

### 2.3. Histochemical Staining

From each tissue block, one paraffin section of 3 µm was cut and mounted on a Superfrost Plus slide (Thermo Fisher Scientific, Waltham, MA, USA). They were dried at 60 °C for 1 h. H&E stains were performed by Ventana HE 600 (Roche Diagnostics, Tucson, AZ, USA) and IHC by Ventana Benchmark Ultra (Roche Diagnostics). SOX10 IHC positivity was visualized with the SOX-10 Rabbit Monoclonal Primary Antibody (SP267; ready-to-use; 32 min; Roche Diagnostics) in combination with either the OptiView DAB IHC Detection Kit (Roche Diagnostics; brown chromogen) or the ultraView Universal Alkaline Phosphatase Red Detection Kit (Roche Diagnostics; red chromogen). Standard settings and regent kits of Ventana Benchmark Ultra (Roche Diagnostics) were used for antigen retrieval (Cc1, 32 min) and endogenous peroxidase blocking (only DAB stains). Immunohistochemical slides were counterstained with Mayer’s hematoxylin and bluing reagent. Internal controls were present in primary melanomas (SOX10 positivity in epidermis).

### 2.4. Scanning

Nanozoomer 2.0HT (Hamamatsu Phototonics KK, Hamamatsu City, Japan) generated WSI of H&E and SOX10 stains at a magnification of 20X (2.04 pixels per µm^2^). 

### 2.5. Regions of Interest

For development purposes, global tumor areas were manually outlined on all digital dual stains in VIS. In the CNN_TB_ test set that included melanoma patients with a BRAF mutation, the same regions initially outlined by a pathologist on routine H&E stains for calculating tumor burden were manually recreated on the associated digital dual stains. In all cases, epidermal regions, skin appendages, and tissue processing artifacts were omitted. To compare procedures, a minimum of three 0.05 mm^2^ squares (512 × 512 pixels at 20X) were automatically identified and outlined by systematic-random sampling (mean area for each analyzed lesion, 0.15 mm^2^; range, 0.06 mm^2^ to 0.24 mm^2^); Figure 1A.

### 2.6. Image Analysis 

**Figure 3:**
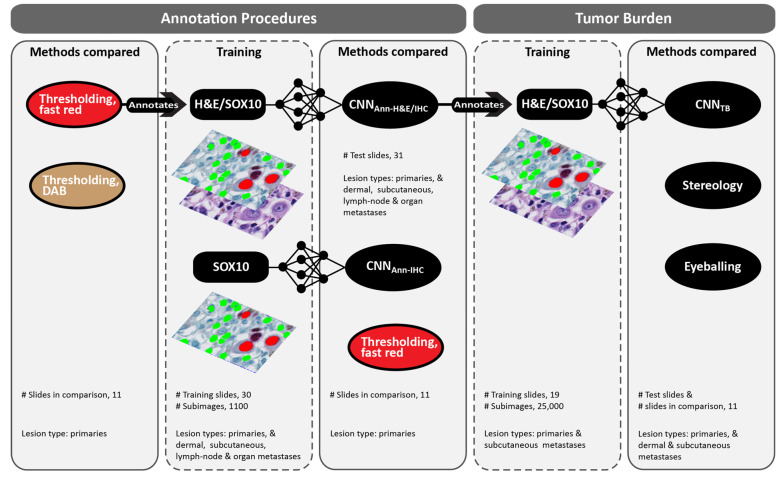
Development of annotation procedures and convolutional neural network for calculating tumor burden (CNN_TB_), and methods compared. Initially, annotation by thresholding using either the red or the brown chromogen of IHC were compared. Thresholding of Fast-Red stains then assisted annotation of digital H&E/IHC dual stains, which was used to train two CNNs for annotation; one with RGB input from IHC (CNN_Ann-IHC_) and one with RGB input from both H&E and IHC (CNN_Ann-H&E-IHC_). The best-performing algorithm annotated the training set of CNN_TB._ Both CNN_TB_ and routine eyeballing of pathologists were compared with stereological counts.

In primary melanomas, the value of the red versus the brown chromogen of SOX10 stains was explored by conventional handcrafted algorithms including thresholding. Then, the utility of using a CNN for annotation (CNN_Ann_) instead of thresholding was explored. This included a CNN that was trained with RGB input of IHC stains (CNN_Ann-IHC_; 3 input bands) and a CNN trained with RGB input from both IHC and H&E (CNN_Ann-H&E/IHC_; 6 input bands). The annotation capabilities of the best-performing application were subsequently examined in metastatic lesions and used for training of the final CNN_TB_; Figure 3.

#### 2.6.1. Subdivision of Lesions for Comparisons and CNN Training, Validation, and Test

To compare the utility of thresholding using either the red (THR_Red_) or the brown chromogen (THR_Brown_), 22 primary melanomas were included (Table 1).

The two CNN_Ann_ were trained on 30 lesions, including eight primary melanomas (Table 1) and two locoregional dermal metastases, in addition to ten resections and ten core needle biopsies of melanoma metastases, that is, seven regional lymph-node metastases and 13 distant metastases to either lymph nodes (*n* = 1), subcutis (*n* = 4), lung (*n* = 2), liver (*n* = 4), or brain (*n* = 2). Its independent test set consisted of 11 primary melanomas (also used for evaluation of THR_Red_; Table 1) and 20 melanoma metastases, that is, five regional and five distant lymph-node metastases in addition to five subcutaneous and five distant organ metastases (lung, *n* = 4, and brain, *n* = 1).

The CNN_TB_ was trained on 13 primary melanomas (Table 1) and six subcutaneous metastases. Its independent test set included six melanomas (Table 1), two locoregional dermal metastases, and three subcutaneous metastases. 

Before any CNN was tested, visual inspection of their performance (validation) was examined on 11 independent primary melanomas (H&E of DAB-stained melanomas) and five independent melanoma metastases, that is, three subcutaneous and two distant lymph-node metastases.

#### 2.6.2. Segmentation by Handcrafted Algorithms 

Thresholding applications were based on preprocessing of the red and blue color bands (chromaticity and contrast), which pin-pointed nuclei of normal cells, remainder tissue, and unstained background, in addition to color deconvolution, which enhanced either the brown or the red staining color of tumor cells.

#### 2.6.3. Segmentation by Neural Network 

Using input images of 512 × 512 pixels, U-nets as presented by Ronneberger et al. [27] were trained in VIS’s Author AI (Visiopharm A/S). This type of net is particular suitable for semantic segmentation of biomedical images [27]. Learning rates, which are based on Adam Optimization [28] in VIS, were set at 1 × 10^−7^ or 1 × 10^−6^, and data augmentation was utilized. Specifics related to U-nets of Visiopharm A/S and training parameters of CNN_Ann-IHC_, CNN_Ann-H&E/IHC_, and CNN_TB_ are displayed in Supplementary Table S1.

The labeled training data for CNN_Ann_ was mainly made with THR_Red_; however, annotations were carefully checked and manually edited if necessary. Additional manual annotations were included to add additional variation to the labeled data. Ultimately, 9174 nuclei of tumor cells (area, 0.80 mm^2^) and 10,698 nuclei of normal cells (area, 0.4 mm^2^) were annotated, in addition to 7711 discrete annotations of the remainder tissue (area, 1.1 mm^2^) and 304 of the unstained background (area, 0.2 mm^2^). Approximately, 1100 subimages/training pairs were included.

By means of this labelled data set, deep learning was initially performed using only RGB input from IHC stains (CNN_Ann-IHC_), but subsequently additional RGB input from H&E stains was included (CNN_Ann-H&E/IHC_). The number of iterations in training was 30,000 for CNN_Ann-IHC_ (28 epochs) and 170,000 for CNN_Ann-H&E/IHC_ (158 epochs).

For CNN_TB_, the labeled training data was made by CNN_Ann-H&E/IHC_ within the entire global tumor outline, which resulted in approximately 25,000 subimages/training pairs. Ultimately, 799,992 nuclei of tumor cells (48 mm^2^) and 421,375 nuclei of normal cells (16 mm^2^) were annotated; yet, nuclei of normal cells were also dilated to form cellular clusters and included again to further adjust CNN_TB_. This resulted in an additional 112,291 normal-cell annotations (area, 7 mm^2^). Moreover, 37,265 discrete annotations for remainder tissue (6 mm^2^) and 7937 for unstained background (32 mm^2^) were included. The CNN_TB_ was trained for 613 iterations, but because no evident progress was observed, the CNN_TB_ at 398,000 iterations was selected (16 epochs). To fine-adjust CNN_TB_, a few manual edits and annotations were made within the labeled training set (<0.015% of all annotations).

All feature maps of neural networks with added mean filters were subsequently classified by thresholding and postprocessing algorithms further enhanced results.

#### 2.6.4. Postprocessing 

Primarily, morphological operations and changes by area or surrounding were utilized. Furthermore, watershed algorithms and polynomial blob filters were applied to separate cellular clusters into individual cells. Similarity of postprocessing algorithms between applications were sought; however, the most optimal composition for each application was the main priority. All final applications were fixed and applied to all related lesions.

#### 2.6.5. Output 

Tumor burden of CNN_TB_ was calculated as a number-based percentage level, that is, the number of tumor-cell nuclei divided by the number of all nuclei within the outlined tumor area. The intensity of the red chromogen was defined as the chromaticity of the red color level. 

### 2.7. Ground-Truth for Procedures of Annotation

Only annotated pixels of an image are visible for the CNN in VIS (Visiopharm A/S); accordingly, all missing or false-negative annotations are irrelevant for the subsequent training of the final CNN. Thus, only the applications’ number of false-positive and true-positive nuclei for both tumor and normal cells were manually annotated and counted in the 0.05 mm^2^-squares of the DAB-stained (*n* = 11) and red-colored melanomas (*n* = 11) and melanoma metastases (*n* = 20).

### 2.8. Ground-Truth Mask for CNN_TB_

Manual annotations were made for all tumor-cell nuclei, normal-cell nuclei, and the remainder tissue in the 0.05 mm^2^-squares of the CNN_TB_ test set (*n* = 11). Accordingly, results of CNN_TB_ were compared with ground-truth masks within 33 pairs of subimages.

### 2.9. Stereology 

In lesions of the CNN_TB_ test set, the same regions initially outlined by a pathologist was recreated on SOX10 stains in VIS. For each tumor, approximately 200 fields of view were identified by systematic-random sampling in which normal cells and tumor cells were manually counted in an unbiased counting frame (30 µm^2^ × 25 µm^2^) at a magnification of 40X. In mean, 243 (range, 97 to 494) tumor cells and 423 (range, 153 to 882) normal cells were counted per lesion. 

### 2.10. Mutant-Allele Frequency by Next-Generation Sequencing 

Mutant-allele frequencies were established as part of routine diagnostics by in-house BRAF-targeted NGS using 10-µm unstained FFPE sections. If the pathologist’s initial eyeballed tumor burden was ≤50%, the unstained section was manually macrodissected (*n* = 6; Table S2) according to the pathologist’s tumor outline on the corresponding H&E slide. Each sample was then subjected to automated genomic DNA extraction using QIAsymphony (QIAGEN, Venlo, The Netherlands). The DNA concentration in each sample was quantified by Qubit (Thermo Fisher Scientific), and the target concentration to perform NGS was 30 ng of DNA. NGS was performed using Ion GeneStudio S5 Prime System with Torrent Suite version 5.12 (Thermo Fisher Scientific) with an average sequencing depth of at least 2000 reads.

### 2.11. Statistics

Precision, which measures how accurate positive predictions are [29], were calculated for all annotation techniques for both tumor-cell nuclei and normal-cell nuclei. Calculations were based on cellular numbers.

Sensitivity, specificity, accuracy, precision, and the weighted-average F1-score were calculated for CNN_TB_ for each individual class and the sum of classes by means of a confusion matrix [29,30]. Calculations were based on the classification of each pixel because cellular numbers are unable to truly quantify the number of true-negative annotations, which also includes the background in image analysis (three classes). 

The 95% confidence intervals (CI) for all proportions were calculated by the Wilson score model [31]. When calculating the 95%CI of CNN_TB_, the number of subimages were used instead of the number of pixels.

Two-sample tests of proportions were utilized to compare precision of annotation techniques, and unpaired *t*-tests investigated the difference between the mean red chromaticity level of primary and melanoma metastases. Both paired *t*-tests and Bland–Altman plots compared stereological counts with the tumor burden of either CNN_TB_ or routine eyeballing. Two-sided *p*-values less than 0.050 were considered statistically significant. 

Statistics and data analysis were made in Stata 12.0 (StataCorp, College Station, TX, USA), RStudio 1.4.1106 (RStudio, PBC, Boston, MA, USA), and MATLAB R2020b (MathWorks, Natick, MA, USA).

## 3. Results

### 3.1. Comparison of Applications for Computer-Assisted Annotation in Primary Melanomas

The performance of applications using thresholding or deep learning are presented in Table 2, and the specific reasons for false-positive annotations are displayed in Table 3 for tumor cells and Table 4 for normal cells.

For thresholding based on either the brown or the red chromogen, the difference in precision was −9.6% (95%CI, −11.2% to −8.0%); *p* < 0.001. A difference predominantly linked to pigmentation falsely annotated as tumor cells (Table 3).

**Figure 4:**
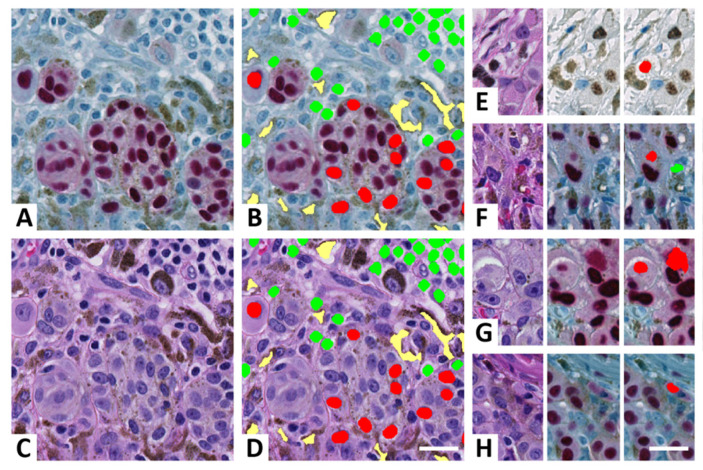
Automated annotation of digital H&E/SOX10 dual-stained melanoma. (**A**) SOX10-stained melanoma cells (red nuclei) and hematoxylin-stained normal cells (blue nuclei). Abundant melanophages are present (blue nucleus and brown melanin in cytoplasm). (**B**) Automated annotation of melanoma cells (red), normal cells (green), and remainder tissue (yellow) by CNN_Ann-H&E-IHC_. (**C**) H&E display of the same tissue area. (**D**) Annotations are directly transferred to H&E. (**E**–**H**) Errors of annotation using either thresholding of DAB stains (**E**) or CNN_Ann-IHC_ on Fast-Red stains (**F**–**H**). By adding RGB input from both SOX10 and H&E in the CNN training, errors (**F**–**H**) were almost eliminated. (**E**) Melanin granules falsely annotated as a tumor-cell nuclei. (**F**) Unspecific red dot falsely annotated as a tumor-cell nucleus and erythrocyte falsely annotated as a normal-cell nucleus. (**G**) False annotation of an unspecific cytoplasmatic reaction and a somewhat dissolved nucleus (left). (**H**) Cytoplasm falsely annotated as tumor-cell nucleus. Scalebars, 25 µm.

To avoid errors caused by pigmentation and to further decrease the number of false-positive events, applications based on deep learning and a red chromogen were established. Initially, deep learning was conducted with IHC stains as the only training input (CNN_Ann-IHC_), but because overfitting quickly occurred and the performance of the neural network was modest (Table 2), information from H&E stains was included (CNN_Ann-H&E/IHC_; Figure 3). The inclusion of H&E to the input of the CNN clearly increased its ability to discriminate IHC colors of nuclei from unspecific staining and the remainder tissue (Figure 4).

The difference in precision between CNN_Ann-H&E/IHC_ and THR_Red_, the two best performing applications, was −0.20% (95%CI, −0.54% to 0.14%; *p =* 0.3) for tumor cells and −1.1% (95%CI, % −2.5% to 0.29%; *p =* 0.2) for normal cells. The difference was not statistically significant, but the number of false-positive annotations were considerably lower for CNN_Ann-H&E/IHC_ than THR_Red_ (Table 3 and Table 4).

Characteristics of labelled objects annotated with CNN_Ann-H&E/IHC_ and THR_Red_ are outlined in Supplementary Table S3 for tumor cells and Supplementary Table S4 for normal cells.

### 3.2. Computer-Assisted Annotation of Metastases 

The CNN_Ann-H&E/IHC_ application was applied to melanoma metastases. Its performance for each metastasis subgroup is displayed in Table 5. 

The difference in precision for primary melanoma versus metastatic melanoma was extremely similar for tumor nuclei (*p =* 1), but markedly reduced for normal-cell nuclei with a mean difference of 8.1% (95%CI, 6.3% to 9.9%); *p* < 0.001. The associated tumor nuclei falsely annotated as normal-cell nuclei (*N* = 109) were almost exclusively caused by absent (*n* = 96) or pale (*n* = 12) SOX10 staining. In addition, one mitotic figure of a tumor cell (blue) was falsely annotated as a nucleus of a normal cell. False-positive tumor-cell annotations were caused by unspecific red dots (*n* = 2; Figure 4F) or unspecific red staining of tumor-cell cytoplasm (*n* = 5; Figure 4G). Only subcutaneous metastases exhibited very few false-positive annotations along with an acceptable annotations rate for normal cells (Table 5).

### 3.3. SOX10 Intensity in Primary Melanomas and Melanoma Metastases

**Figure 5:**
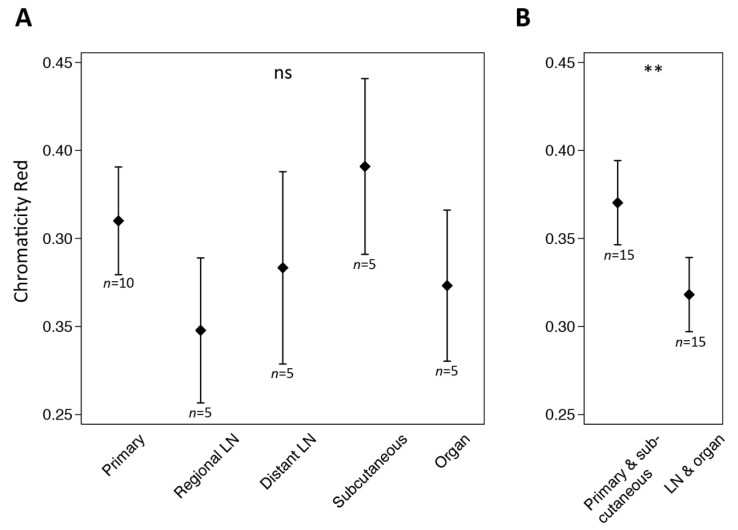
Mean red chromaticity level of annotated tumor cells with 95% confidence intervals for (**A**) each individual tumor type (primary melanomas and metastases of regional and distant lymph nodes (LN), subcutis, and organs) and (**B**) tumors of the skin (primary melanomas and subcutaneous metastases) versus metastatic tumors of LN and organs. (ns, not significant; ** very significant).

The high number of pale or SOX10-negative tumor cells in lymph-node and distant organ metastases, led to an analysis of the red-color level (red chromaticity) in primary melanomas and melanoma metastases (Figure 5).

By combining primary melanomas and subcutaneous metastases (*n* = 15), the mean red-chromaticity level was 0.37 (95%CI, 0.35 to 0.39) while the mean of organ and lymph-node metastases (*n* = 15) was 0.32 (0.30 to 0.34); *p =* 0.002 (Figure 5B). When analyzed separately, the intensity level of SOX10 was also significantly higher for primary melanomas (*n* = 10; *p =* 0.02) and for subcutaneous metastases (*n* = 5, *p* = 0.002) when compared with both organ and lymph-node metastases (*n* = 15).

### 3.4. Performance of Neural Network for Calculating Tumor Burden

Because our procedure for IHC-verified annotation only seemed feasible for primary melanomas and subcutaneous metastases, only these lesion types were included in the development and test of CNN_TB_. 

For all three classes, the weighted-average F1-score of CNN_TB_, in addition to precision and sensitivity, was 88.8% (95%CI, 79.0% to 94.4%). The associated accuracy was 92.6% (95%CI, 83.6% to 96.8%) and its specificity 94.4% (95%CI, 86.0% to 97.9%). The segmentation performance for each individual class (tumor-cell nuclei, normal-cell nuclei, and background) are displayed in Table 6.

**Figure 6:**
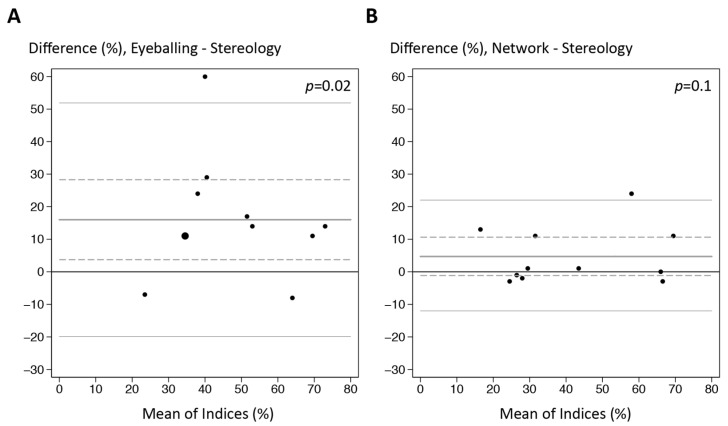
Bland–Altman plots that compare the tumor burden of stereological counts with either the pathologist’s eyeballing of routine diagnosis (**A**) or the automated calculation of the convolutional neural network CNN_TB_ (**B**). The 95% limits of agreement (thin grey lines) and the mean difference (thick grey line) with associated 95% confidence intervals (hatched grey lines) are shown. Enlarged dot in (**A**) resembles two samples with the same percentage levels.

The tumor burden of both routine diagnosis and CNN_TB_ are compared with stereological counts in Figure 6A,B, respectively. For each case included in the test set, the tumor burden of stereology, mutant-allele frequency, eyeballing, and CNN_TB_ are displayed in Supplementary Table S2. 

Because tumor cells of regional lymph-node resections and skin lesions shared a similar appearance, CNN_TB_ was subsequently tested on lymph-node metastases with a BRAF mutational status (*n* = 5). The tumor burden of lymph nodes for both routine diagnosis and CNN_TB_ are compared with stereological counts in Supplementary Figure S1.

## 4. Discussion

An automated, IHC-supervised annotation technique with high precision was developed for melanoma and subcutaneous metastases by means of digital H&E/SOX10 dual stains with a red chromogen. For these lesion types, the associated annotated training set with only a few manual annotations for fine-tuning (<0.015%) generated a high-performing CNN for calculating tumor burden, which was superior to the pathologists’ routine practice. To our knowledge, this is the first study to present a CNN for calculating tumor burden with lesions relevant for a clinical setting.

In previous studies of IHC-supervised learning, only the brown chromogen DAB for annotation of digital H&E/IHC stains has been utilized [20,21]; however, the brown color is less suitable for melanocytic lesions because they often include various amounts of melanin; especially within melanophages (Figure 4A,E). In accordance, our study demonstrated a highly significant (*p* < 0.001) increase in the technique’s performance by using a red instead of a brown chromogen in primary melanomas. Moreover, the annotation rate for THR_Red_ was approximately twice as high compared with THR_Brown_, which indicates that procedures of image analysis were simplified, i.e., less indecisive annotations were excluded by final postprocessing algorithms. All false-positive annotations in DAB stains were related to melanin falsely annotated as tumor cells (Table 3). Because the study included slides from three previous studies [13,25,26], the lesions compared were unmatched; however, both subgroups included similar amounts of pigmentation. 

When developing THR_Red_, repetitive errors were also observed; that is, unspecific SOX10 reactions unrelated to tumor nuclei (Table 3 and Figure 4F–H). Especially, unspecific red dots were falsely annotated as tumor cells. In some cases, these dots may have represented somewhat dissolved nuclei or lost caps, but often their appearance was very indistinct on H&E stains (Figure 4G). They were thus considered false-positive and undesirable for CNN_TB_ training. To avoid these errors, utility of a CNN for annotation was explored with RGB input from only IHC and from both IHC and H&E.

Even though they shared an identical labelled training set, the performance of CNN_Ann-H&E/IHC_ was highly superior to CNN_Ann-IHC_ (Table 2). CNN_Ann-IHC_ was, in addition, prone to overfitting, and more annotations were of questionable quality. Conceivably, CNN_Ann-IHC_ could have been improved by additional training, but a considerable number of new annotations seemed necessary. Importantly, CNN_Ann-H&E/IHC_ almost eliminated all mistakes related to unspecific SOX10 reactions and skewed alignments (Table 3 and Table 4). The additional information from H&E stains thus seems very valuable to include in a CNN for SOX10 detection and possibly for detection of many other IHC markers as well.

When comparing CNN_Ann-H&E/IHC_ and THR_Red_, the difference in precision was not statistically significant, but the number of false-positive annotations were considerably lower for CNN_Ann-H&E/IHC_ (Table 3 and Table 4) than THR_Red_, although their annotation rates were somewhat the same (Table 2). When manually examined, errors also seemed less apparent for CNN_Ann-H&E/IHC_ compared with THR_Red_. 

In a test set of new and independent lesions, our precision of CNN_Ann-H&E/IHC_ was close to 100% for annotation of both tumor cells and normal cells. In the study of Jackson et al., the specificity of their SOX10 annotation was 86% in subimages (10% of master set) of the same lesions that also were included in the training set (90% of master set) [21].

Initially, a working hypothesis was that CNN_Ann_ would facilitate a larger variation in the appearance of the annotated cells compared with thresholding, but this seemed unjustified from study data (Tables S3 and S4). On the contrary, the appearance of annotated cells seemed somewhat similar for both procedures (Tables S3 and S4); yet, differences in area and form factor were statistically significant for tumor cells (Table S3). As opposed to chromaticity, these features could, however, have been influenced by postprocessing algorithms of the study. Correspondingly, because postprocessing subsequently alters the result of segmentation based on various characteristics of the objects of interest (e.g., size, shape, color), some manual annotations are possibly always necessary to include additional variation in the labelled training set. 

Given a slightly better performance and because applications based on CNN, generally, are less reliant on stain quality and IHC standardization compared with simple thresholding, CNN_Ann-H&E/IHC_ was subsequently applied to metastases and the training set of CNN_TB_. 

In metastases, a high number of SOX10-negative tumor cells was observed in lymph-node and organ metastases. Furthermore, the appearance of tumor cells was very different in core needle biopsies compared with resections. Accordingly, the performance of CNN_Ann-H&E/IHC_ was low for these lesion types. In addition, very few annotations were often created, especially in organ metastases (Table 5). The annotation technique thus seemed less useful for such lesion types, unless time-consuming manual corrections were to be performed. Other studies have also demonstrated that the percentage level of SOX10-positive tumor cells may vary considerably in both primary and metastatic melanoma [32,33,34,35]. Although our study mostly detected SOX10-negative cells in metastatic melanoma, presence of negative tumor cells is an unavoidable limitation of the proposed SOX10 annotation technique. Consequently, the general SOX10 positivity of the tumor should be checked before inclusion in the training set. In addition, some normal cells may also display SOX10 positivity, e.g., Schwann cells [36], cells of eccrine sweat glands [37], and mast cells that may be very abundant in the periphery of some melanomas [36,38]. Eccrine sweat glands are, however, easily recognized during the initial tumor outline, in which they may be manually omitted or an automated procedure for exclusion may possibly be developed.

This is one of the first studies to compare the intensity level of SOX10-positive cells in primary melanomas and various types of metastatic melanoma by image analysis (chromaticity red). A highly statistically significant difference was demonstrated for lesions of the skin compared with lymph-node and organ metastases (Figure 5B), even though our sample size was small. These results are in line with a large study by Agnarsdóttir et al., in which an automated intensity level of SOX10 was compared for melanomas (*n* = 106) and their related metastases (*n* = 45). In addition, the study demonstrated an inverse relationship between Ki67 and SOX10; that is, low SOX10 intensity was associated with high proliferation [35]. Because metastases, in general, display a higher proliferative potential than primary melanomas [39,40], a low SOX10 expression of metastases seems justified. In the study of Agnarsdóttir et al., 81% of the primary tumors displayed SOX10 positivity in more than 75% of tumor cells, and 3% displayed cellular positivity in less than 25% of tumor cells. Numbers for metastases were unreported [35]. In the study of Bakos et al., the intensity level of SOX10-positive cells was scored in primary melanomas and cutaneous and subcutaneous metastases by conventional microscopy. Though more of their SOX10-positive metastases were weakly stained (10 of 10; 100%) compared with primary melanomas (15 of 21; 71%), approximately half of both lesion types were SOX10-negative [33]. In the study of Mohamed et al., all primary melanomas (*n* = 109) and melanoma metastases to the brain (*n* = 11) were SOX10 positive (with >50% of cells being positive), and all lesions achieved a high SOX10 intensity score by microscopy [32]. In the study of Shakhova et al., 100% of all primary melanomas were marked by SOX10, and in 85% of all samples (*n* = 48), more than 90% of cells were SOX10 positive. In their metastatic samples from various anatomic sites (*n* = 130) except subcutis, 13% exhibited either very restricted or no nuclear SOX10 staining [34]. The overall differences between studies may reflect the use of different SOX10 IHC protocols. Moreover, high intra and interobserver variability is often associated with manual IHC intensity scores [8], which may be reduced by image analysis [41]. 

Overall, the training set of CNN_Ann-H&E/IHC_ yielded a CNN_TB_ with high performance in lesions of the skin, i.e., the sensitivity and specificity were 88% and 94%, respectively, for the classification of tumor-cell nuclei, normal-cell nuclei, and the remainder background. Yet, when looking at the classes individually, the sensitivity for detecting normal-cell nuclei and the precision for detecting tumor-cell nuclei was rather low for CNN_TB_, alongside the F1 score (Table 6). This was consistent with manual inspections of the results, that is, many normal-cell nuclei were falsely annotated as tumor cells, which increases both the number of false-negative entities when calculating the sensitivity for normal cells and the number of false-positive entities when calculating the precision for tumor cells. For tumor cells, the most frequent error was large nucleoli or small hyperchromatic cells annotated as normal-cell nuclei. Furthermore, elongated stromal-cell nuclei with large nucleoli and nuclei of macrophages caused inaccuracies in our study. In the only lesion that differed markedly from stereology (ID 7; Table S2), high resemblance between normal and tumor cells was observed. Furthermore, many neutrophile and eosinophile granulocytes were present, and the granulocytes with many lobes were often falsely classified as tumor cells. This could possibly have been corrected with further training of CNN_TB_. By coincidence, the number of granulocytes was fairly limited in the training set of CNN_TB_. Difficulties in separating clusters of normal cells were also observed for CNN_TB_ (Figure 1E), even though many postprocessing algorithms were employed to obtain a correct cellular count.

To optimize CNN_TB_, resources of open source, including other network types and designs, could be explored, and possibly a combination of IHC-verified and weakly supervised learning may prove beneficial. Furthermore, to fully evaluate the performance of IHC-verified annotation, it should be compared with weakly supervised models, both in terms of hands-on-time and performance of the final neural net.

Yet, melanomas are characterized by their ability to present a diverse array of cytomorphologic features, in which size, shape, and color of their nuclei may vary considerably both within and between tumors. For instance, melanomas may be composed of large pleomorphic cells, small cells, spindle cells, and nuclei may show bi- or multi-nucleation, lobation, inclusions, grooving, and angulation [19]. Accordingly, they may in some cases share similar features of normal-cell nuclei, which also was evident in our study. Immunohistochemistry thus seems necessary to accurately differentiate nuclei in network training. Consequently, a flawless differentiation of normal-cell and tumor-cell nuclei seems an unrealistic task, even though it is possible to continuously train a CNN_TB_ to increase its performance. In addition, when presented with only the nucleus, the task becomes even more complex for a CNN; hence, information from the remainder tissue, e.g., associated cytoplasm and cellular architecture, is often indispensable for the pathologist to render a correct diagnosis. Accordingly, it seems better to annotate clusters of cells instead of single cells, but consequently, it becomes difficult to separate each nucleus afterwards, which is necessary when calculating the tumor burden. Jackson et al. also states that their results were imperfect, that is, several foci of lymphocytes as well as occasional keratinocytes were falsely annotated as tumor cells [21]. In our study, epidermis was deliberately excluded from all analyses given the resemblance between keratinocytes and some tumor cells, and because epidermis may include normal SOX10-positive melanocytes. 

We manually outlined the dermal tumor compartment excluding adnexae and tissue artifacts in this study, but an automated stratification of the regions of interest is possibly feasible to create a fully automated procedure for calculating tumor burden on H&E stains. Epidermis of IHC has previously been automatically identified by handcrafted algorithms [42], but neural nets certainly hold the potential to identify the different layers of the skin based on H&E stains, which may be useful in many research projects and in future diagnostic settings where the location of a biomarkers often is important.

Although errors were apparent from the CNN_TB_ test set, its calculation of tumor burden was highly superior to the pathologist’s estimate (Figure 6 and Table S2). In contrast to CNN_TB_, the difference between the pathologists and stereology was statistically significant (Figure 6A). Typically, the estimates of the pathologists were higher than the counts of stereology. In some cases, this may be explained by the enlarged nuclei of tumor cells, which may produce a large tumor area but not necessarily a high tumor burden (Figure 1), which is based on cellular numbers. Our low accuracy of the pathologists is in line with previous reports of low reproducibility and low accuracy among pathologists eyeballing tumor burden [10,11]. Lhermitte et al. states that 38% of their study samples with a low tumor content (<20%) were overestimated by pathologists and thus associated with a higher risk of a false-negative BRAF result [11].

While the performance of CNN_TB_ was satisfactory in this study, our results need to be validated in a larger, independent test set in order to use CNN_TB_ in a routine setting. CNN_TB_ was, however, only developed for primary melanomas and metastases of the skin; yet, results for lymph-node resections were promising (Figure S1). The clinical practice guidelines, however, recommend using the metastasis if available and suitable for molecular analysis; otherwise, the primary tumor may be analyzed [43]. Some advocate for the use of the primary tumor, but intertumoral heterogeneity of BRAF between a patient’s primary and subsequent metastatic lesion is still discussed [44,45], and a metanalysis has proposed a possible discrepancy rate of approximately 10% [46]. Thus, our CNN_TB_ is, currently, only useful in a subset of lesions in a clinical setting. To include other types of metastases, additional neural networks may be developed, possibly for each individual organ site by manual annotations. This is, however, a very time-consuming and difficult task when tumor cells frequently are SOX10 negative.

An advantage of our study was that the count of stereology (the gold standard) could be compared with the mutant-allele frequency of NGS. Though it remains discussed whether a BRAF mutation is homozygous or heterozygous [47,48], the two numbers seemed in range (Table S2). The regions of interest analyzed in each method may, however, have varied slightly. Of particular importance, some pathologists may have been unaware of the general limitations associated with macrodissection. Consequently, their tumor burden may have been based on a very detailed tumor outline, which often is very difficult to recapitulate for the technician in the subsequent dissection of the tumor area. Additional and redundant areas with normal cells may thus have been included in the molecular analysis, which especially affects the allele frequency of lymph-node metastases that often include many lymphocytes (Figure 1). 

In our study, the evaluations of image analysis were based on a minimum of three fairly small subfields (Figure 1A). This was done to create a very accurate ground truth within a reasonable time-frame. Although these fields only represented a small fraction of the entire tumor, they included approximately 3000 cells and 25,000,000 pixels in the CNN_TB_ test set. 

One general disadvantage of our digital H&E/SOX10 procedure was that the coverslip was quite difficult and time-consuming to remove for the technician. Yet, fairly novel whole slide scanners that are able to scan without coverslips may solve this issue, and they possibly hold great promise for the future development and application of digital multiple stains in pathology.

## 5. Conclusions

By means of digital H&E/SOX10 dual stains with a red chromogen, a large annotated H&E training set with high quality was created within a reasonable timeframe for primary melanomas and metastases of the skin. For these lesion types, the training set generated a high-performing CNN for calculating tumor burden, which was superior to the pathologists’ routine eyeballing. Yet, due to low or missing tumor-cell SOX10 positivity, advantages of the annotation technique were limited in lymph-node and organ metastases.

## Figures and Tables

**Table 1:** Histopathological Characteristics of Included Melanomas.

Feature	Annotation			CNN_TB_	
	THR_brown_, *n* = 11	CNN_Ann_ Training, *n* = 8	THR_red_ and CNN_Ann_ Test, *n* = 11	Training, *n* = 13	Test, *n* = 6
Mean Breslow Thickness (mm)	3.79	3.67	3.27	2.97	4.46
Ulcerated Lesions, *n* (%)	7 (64)	2 (25)	4 (36)	4 (31)	2 (33)
Histopathological Subtype, *n* (%)					
Superficial Spreading	6 (55)	5 (63)	8 (73)	9 (69)	4 (67)
Nodular	4 (36)	2 (25)	2 (18)	2 (15)	2 (33)
Lentigo Maligna Melanoma	1 (9)	0	1 (9)	1 (8)	0
Unclassified	0	1 (13)	0	1 (8)	0

Abbreviations: THR_brown_, thresholding of DAB stains; THR_red_, thresholding of Fast-Red stains; CNN_Ann-IHC_, convolutional neural network for annotation trained with only immunohistochemistry; CNN_Ann-H&E/IHC_, convolutional neural network for annotation trained with both H&E stains and immunohistochemistry; CNN_TB,_ convolutional neural net for calculating tumor burden.

**Table 2:** Performance of Annotation Procedures in Primary Melanomas.

Label Type	APP Type	Precision (95%CI), %	No. of FP	No. of TP	Annotation Rate (labels/mm^2^)
Tumor-Cell Annotation	THR_brown_	89.9 (88.2 to 91.3)	146	1298	962
	THR_red_	99.5 (99.1 to 99.7)	15	2843	1739
	CNN_Ann-IHC_	97.5 (97.0 to 98.0)	92	3597	2245
	CNN_Ann-H&E/IHC_	99.7 (99.4 to 99.9)	7	2468	1506
Normal-Cell Annotation	THR_red_	98.1 (96.7 to 98.9)	12	626	388
	CNN_Ann-IHC_	88.8 (84.0 to 92.3)	25	199	136
	CNN_Ann-H&E/IHC_	99.2 (97.7 to 99.7)	3	373	229

Abbreviations: APP, application; CI, confidence interval; FP, false positive; TP, true positive; THR_brown_, thresholding of DAB stains; THR_red_ thresholding of Fast-Red stains; CNN_Ann-IHC_, convolutional neural network for annotation trained with only immunohistochemistry; CNN_Ann-H&E/IHC_, convolutional neural network for annotation trained with both H&E stains and immunohistochemistry.

**Table 3:** Reasons for False-Positive Tumor-Cell Annotations in Primary Melanomas.

APP Type	No. of False-Positive Labels (%)				
	Pigmentation	Skewed Alignment	Cytoplasmatic SOX10 Reaction	Unspecific Red Dot	Only Part of Nucleus Edge Detected
THR_brown_	146 (100)	0	0	0	0
THR_red_	0	0	4 (27)	11 (73)	0
CNN_Ann-IHC_	0	14 (15)	34 (37)	36 (39)	8 (9)
CNN_Ann-H&E/IHC_	0	1 (14)	3 (43)	3 (43)	0

Abbreviations: APP, application; THR_brown_, thresholding of DAB stains; THR_red_, thresholding of Fast-Red stains; CNN_Ann-IHC_, convolutional neural network for annotation trained with only immunohistochemistry; CNN_Ann-H&E/IHC_, convolutional neural network for annotation trained with both H&E stains and immunohistochemistry.

**Table 4:** Reasons for False-Positive Normal-Cell Annotations in Primary Melanomas.

APP Type	No. of False-Positive Labels (%)				
	Skewed Alignment	SOX10-Negative Tumor Cell	Pale SOX10-Positive Cell	Dark Blue Cytoplasm	Mitotic Figure within Tumor Nest
THR_red_	1 (8)	11 (92)	0	0	0
CNN_Ann-IHC_	6 (24)	0	2 (8)	16 (64)	1 (4)
CNN_Ann-H&E/IHC_	0	3 (100)	0	0	0

Abbreviations: APP, application; THR_red_, thresholding of Fast-Red stains; CNN_Ann-IHC_, convolutional neural network for annotation trained with only immunohistochemistry; CNN_Ann-H&E/IHC_, convolutional neural network for annotation trained with both H&E stains and immunohistochemistry.

**Table 5:** Performance of Convolutional Neural Network for Annotation (CNN_Ann-H&E/IHC_) in Metastases.

Label Type	Site of Metastasis	Precision (%, 95%CI)	No. of FP	No. of TP	Annotation Rate (Labels/mm^2^)
Tumor-Cell Annotation	All (*N* = 20)	99.7 (99.3 to 99.8)	7	2177	614
	Regional lymph node (*n* = 5)	99.8 (98.6 to 100)	1	405	312
	Distant lymph node (*n* = 5)	99.1 (96.8 to 99.8)	2	219	340
	Subcutis (*n* = 5)	99.6 (99.1 to 99.9)	4	1127	1392
	Lung (*n* = 4)	100 (99.1 to 100)	0	421	666
	Brain (*n* = 1)	100 (56.6 to 100)	0	5	31
Normal-Cell Annotation	All (*N* = 20)	91.1 (89.6 to 92.8)	109	1113	343
	Regional lymph node (*n* = 5)	95.9 (93.5 to 97.4)	17	393	315
	Distant lymph node (*n* = 5)	92.1 (89.5 to 94.1)	43	499	833
	Subcutis (*n* = 5)	100 (97.9 to 100)	0	178	219
	Lung (*n* = 4)	62.5 (30.6 to 86.3)	3	5	13
	Brain (*n* = 1)	61.3 (52.4 to 69.6)	46	73	733

Abbreviations: CI, confidence interval; FP, false positive; TP, true positive; CNN_Ann-H&E/IHC_, convolutional neural network trained with both H&E stains and immunohistochemistry.

**Table 6:** Segmentation Performance of Neural Network (CNN_TB_) for Each Individual Class.

Metric with 95%CI, %	Tumor Nuclei	Normal Nuclei	Background
Sensitivity/recall	84.0 (73.3 to 90.9)	54.4 (42.5 to 65.8)	95.5 (87.5 to 98.5)
Specificity	94.3 (85.8 to 97.8)	98.7 (92.2 to 99.8)	79.6 (68.4 to 87.6)
Accuracy	93.1 (85.9 to 97.8)	93.0 (84.2 to 97.1)	91.5 (82.3 to 96.1)
Precision	66.6 (54.5 to 76.8)	86.4 (76.1 to 92.7)	93.4 (84.7 to 97.3)
F1 score	74.3 (62.6 to 83.3)	66.8 (54.8 to 77.0)	94.4 (86.0 to 97.7)

Abbreviations: CNN_TB,_ convolutional neural net for calculating tumor burden; CI, confidence interval.

## Data Availability

The data presented in this study are available on request from the corresponding author.

## Supplementary Materials

The following supporting information can be downloaded at: https://www.mdpi.com/article/10.3390/ijerph192114327/s1, Table S1: Input, network details, and training parameters of the study’s U-nets; Table S2: Tumor Burden of Test Set for Stereology, Mutant Alleles, Eyeballing, and Neural Net; Table S3: Characteristics of Tumor Labels Detected by Thresholding or Neural Net; Table S4: Characteristics of Normal Labels Detected by Thresholding or Neural Net; Figure S1: Bland–Altman for plots lymph-node metastases. Reference [49] is cited in the supplementary materials.
