# Supplementary material for: Computer-Assisted Annotation of Digital H&E/SOX10 Dual Stains Generates High-Performing Convolutional Neural Network for Calculating Tumor Burden in H&E-Stained Cutaneous Melanoma
Source: Int J Environ Res Public Health. 2022 Nov 2;19(21):14327. doi: 10.3390/ijerph192114327 (PMC9654525; doi:10.3390/ijerph192114327)
Supplement: Supplementary file 1 [file ijerph-19-14327-s001.zip › ijerph-1906311-supplementary.pdf]

Table S1. Input, network details, and training parameters of the study's U-nets

	$CNN_{Ann-IHC}$	$CNN_{Ann-H\&E/IHC}$	CNN_{TB}
Input			
Channels (number of channels)	RGB of IHC (3)	RGB of IHC and H&E (6)	RGB of H&E (3)
Image Size	512 x 512 pixels	512 x 512 pixels	512 x 512 pixels
Preprocessing	Mean subtraction per input dimension, $x' = x - mean(x)$ where x is the original value and x' the new value		
Number of Subimages	1100	1100	25,000
Number of Annotations	27,887	27,887	1,378,860
Network Design			
Architecture	U-net of Ronnenberger et al. [27]		
Activation Function	Rectified Linear Units (ReLu)		
Network Parameters			
Weight Initialization	Pretrained weights (ImageNet) of the first 2 layers, otherwise random values with He-Normal initializer [49]	Random values with He-Normal initializer [49]	Pretrained weights (ImageNet) of the first 2 layers, otherwise random values with He-Normal initializer [49]
Freeze Depth	2	0	2
Training Parameters			
Learning Rate (Adam Optimization) [28]	$1 \cdot 10^{-7}$	$1 \cdot 10^{-6}$	$1 \cdot 10^{-6}$
Loss Function	Cross entropy, $Loss_{CrossEntropy} = \sum_{i \in classes} y_{label_i} \cdot logP_i$ where y_{label} is the ground truth label and P the probability of the i-th class		
Loss Weighting	No	No	Class constant weighting to balance size of labelled data
Minibatch size	1	1	1
Augmentation	Rotation (90°, 180°, 270°), flipping (vertical and horizontal), and brightness and contrast perturbations (probability parameter, 0.5)	Rotation (90°, 180°, 270°) flipping (vertical and horizontal), and brightness and contrast perturbations, H&E stain specific perturbations, including perturbations of hue and saturation (probability parameter, 0.5)	Rotation (90°, 180°, 270°) flipping (vertical and horizontal), and brightness and contrast perturbations, H&E stain specific perturbations, including perturbations of hue and saturation (probability parameter, 0.5)
Iterations (epochs)	30,000 (28)	170,000 (158)	398,000 (16)

Abbreviations: $CNN_{Ann-IHC}$, convolutional neural network for annotation trained with only immunohistochemistry; $CNN_{Ann-H\&E/IHC}$, convolutional neural network for annotation trained with both H&E stains and immunohistochemistry; CNN_{TB}, convolutional neural network for calculating tumor burden.

Table S2. Tumor Burden of Test Set for Stereology, Mutant Alleles, Eyeballing, and Neural Net

ID	Lesion Type	Macro-dissected	Mutant Alleles†, %	Stereology*, %	Eyeballing, %	CNN_{TB}, %
1	Primary melanoma	Yes	16 or 32	29	40	27
2	Primary melanoma	No	31 or 62	43	60	44
3	Primary melanoma	No	34 or 68	68	60	65
4	Primary melanoma	No	20 or 40	27	20	26
5	Primary melanoma	Yes	14 or 28	29	40	30
6	Primary melanoma	Yes	15 to 30	28	50	23
7	Locoregional dermal metastasis	No	26 to 52	46	60	70
8	Locoregional dermal metastasis	Yes	17 to 34	26	55	37
9	Subcutaneous metastasis	No	75	64	75	75
10	Subcutaneous metastasis	Yes	5 or 10	10	70	23
11	Subcutaneous metastasis	Yes	57	66	80	66

† The first number of the mutant-allele frequency holds true if the tumor mutation is homozygous and the second number if the tumor mutation is heterozygous [47, 48].

* Green numbers differ 0% to 8% from stereology, yellow numbers 11% to 17%, and red numbers 22% to 60%.

Abbreviations: CNN_{TB}, convolutional neural net for calculating tumor burden

Table S3. Characteristics of Tumor Labels Detected by Thresholding or Neural Net

Metric		THR_{red}	$CNN_{Ann\text{-}H\&E/IHC}$	*p*-value*
Red Chromaticity	Median (range)	0.37 (0.26 to 0.50)	0.39 (0.20 to 0.57)	0.35
	SD	0.038	0.059	
Form Factor†	Median (range)	0.79 (0.15 to 0.95)	0.85 (0.24 to 0.95)	0.01
	SD	0.11	0.09	
Area of Cell (µm²)	Median (range)	82 (9 to 223)	44 (20 to 225)	0.001
	SD	37	16	

* Paired *t*-test

† $F = \frac{4\pi \cdot A_{label}}{{P_{label}}^2}$ were A_{label} is the area of the labelled cell and P_{label} its perimeter.

Abbreviations: THR_{red}, thresholding of Fast-Red stains; $CNN_{Ann\text{-}H\&E/IHC}$, convolutional neural network for annotations trained with both H&E stains and immunohistochemistry; SD, standard deviation

Table S4. Characteristics of Normal Labels Detected by Thresholding or Neural Net

Metric		THR_{red}	$CNN_{Ann\text{-}H\&E/IHC}$	*p*-value*
Blue Chromaticity	Median (range)	0.45 (0.41 to 0.52)	0.45 (0.39 to 0.55)	0.21
	SD	0.017	0.031	
Form Factor†	Median (range)	0.88 (0.41 to 0.95)	0.86 (0.62 to 0.94)	0.14
	SD	0.074	0.062	
Area of Cell (µm²)	Median (range)	40 (8 to 72)	36 (12 to 67)	0.60
	SD	11	9	

* Paired t-test

† $F = \frac{4\pi \cdot A_{label}}{{P_{label}}^2}$ were A_{label} is the area of the labelled cell and P_{label} its perimeter.

Abbreviations: THR_{red}, thresholding of Fast-Red stains; $CNN_{Ann\text{-}H\&E/IHC}$, convolutional neural network for annotation trained with both H&E stains and immunohistochemistry; SD, standard deviation

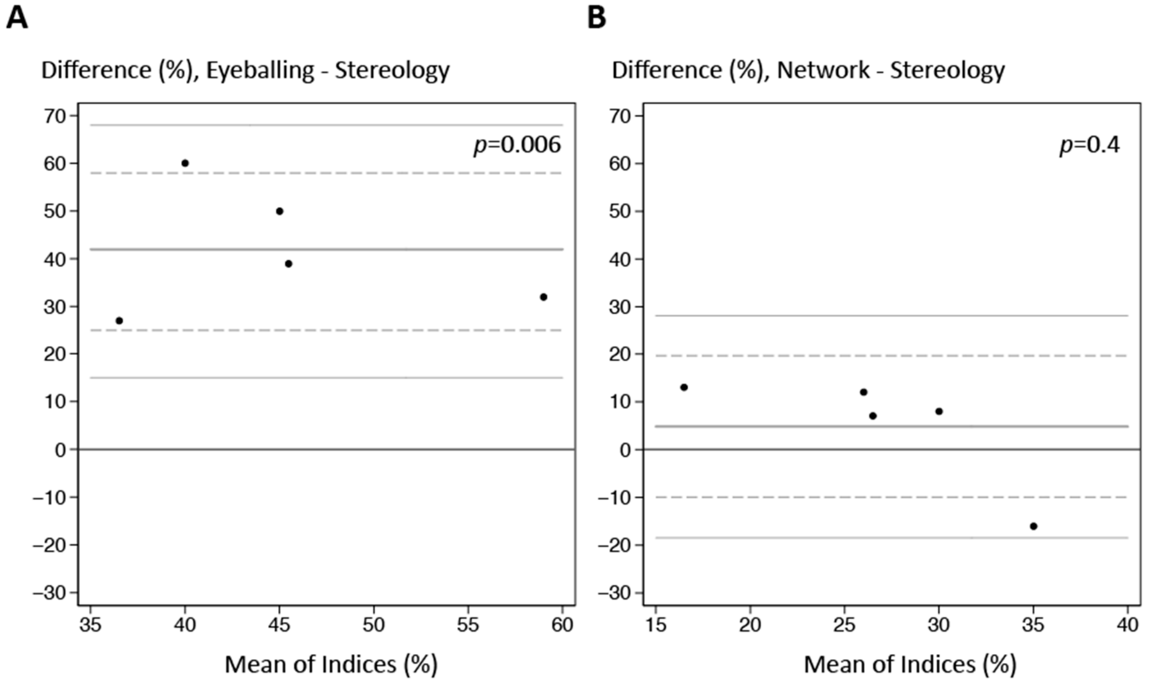

Figure S1. Bland-Altman plots for lymph-node metastases that compare the tumor burden of stereological counts with either the pathologist's eyeballing of routine diagnosis (**A**) or the automated calculation by the convolutional neural network CNN_{TB} (**B**). The 95% limits of agreement (thin grey lines) and the mean difference (thick grey line) with associated 95% confidence intervals (hatched grey lines) are shown.
